# Network resonance and the auditory steady state response

**DOI:** 10.1038/s41598-024-66697-4

**Published:** 2024-07-22

**Authors:** Teryn D. Johnson, Austin J. Gallagher, Seana Coulson, Lara M. Rangel

**Affiliations:** Department of Cognitive Science, University of California San Diego, La Jolla, 92093 USA

**Keywords:** Auditory steady state response, Electroencephalography, Local field potential, Resonance, Neural oscillations, Neuroscience, Physiology, Psychology, Biomarkers

## Abstract

The auditory steady state response (ASSR) arises when periodic sounds evoke stable responses in auditory networks that reflect the acoustic characteristics of the stimuli, such as the amplitude of the sound envelope. Larger for some stimulus rates than others, the ASSR in the human electroencephalogram (EEG) is notably maximal for sounds modulated in amplitude at 40 Hz. To investigate the local circuit underpinnings of the large ASSR to 40 Hz amplitude-modulated (AM) sounds, we acquired skull EEG and local field potential (LFP) recordings from primary auditory cortex (A1) in the rat during the presentation of 20, 30, 40, 50, and 80 Hz AM tones. 40 Hz AM tones elicited the largest ASSR from the EEG acquired above auditory cortex and the LFP acquired from each cortical layer in A1. The large ASSR in the EEG to 40 Hz AM tones was not due to larger instantaneous amplitude of the signals or to greater phase alignment of the LFP across the cortical layers. Instead, it resulted from decreased latency variability (or enhanced temporal consistency) of the 40 Hz response. Statistical models indicate the EEG signal was best predicted by LFPs in either the most superficial or deep cortical layers, suggesting deep layer coordinators of the ASSR. Overall, our results indicate that the recruitment of non-uniform but more temporally consistent responses across A1 layers underlie the larger ASSR to amplitude-modulated tones at 40 Hz.

## Introduction

Steady state responses arise when periodic stimuli in the environment evoke stable, stereotyped activity in sensory networks. These responses are highly rhythmic, such that predictable voltage fluctuations that match the frequency of the periodic stimulus arise and vary little in phase and amplitude^1^. For example, a click train delivered at the rate of 20 clicks per second elicits a 20 Hz auditory steady state response (ASSR) in the electroencephalogram (EEG). The ASSR is variously known as the auditory steady-state evoked potential (SSEP), the envelope following response (EFR), and the amplitude-modulation following response (AMFR). Due in part to the ease of conducting non-invasive EEG recordings, the ASSR has emerged as a potential biomarker for altered neural function in schizophrenia, autism, bipolar disorder, attention-deficit/hyperactivity disorder, and Alzheimer’s disease^2–11^. One intriguing aspect of the ASSR is the relationship between the amplitude modulation (AM) frequency (i.e., the rate of fluctuations in the sound envelope) and the magnitude of the ASSR^12–14^. The largest ASSR in humans typically occurs in response to 40 Hz AM^14,15^, and while there is an overall reduction in the ASSR across multiple AM frequencies in patient populations, the reduction in the ASSR to 40 Hz AM is particularly pronounced in patients with schizophrenia^3,16–20^. The utility of the ASSR as a translational tool, however, is compromised in part due to uncertainties regarding the mechanisms that underlie its generation.

There have been several competing explanations for why 40 Hz AM is the best modulation frequency for the ASSR. An early account is that transient middle latency responses in A1 superimpose optimally at the 25 ms inter-stimulus interval corresponding to 40 Hz^21–24^. While this *superposition* account provides a good explanation of the enhanced ASSR to 40 Hz AM, it fails to predict differences in the amplitude of steady state responses to AM frequencies outside of 40 Hz^25,26^. An alternative *oscillatory* account is that local circuits within A1 generate an intrinsic 40 Hz rhythm, giving rise to spontaneous oscillations at that frequency^27,28^. On such an account, neural resonance at 40 Hz underlies the timing of both transient and rhythmic responses in A1. Accordingly, A1 exhibits both spontaneous and stimulus-induced gamma band oscillations near 40 Hz^29–32^. These oscillations have been shown to strongly influence the timing of A1 activity, and to align temporally to the onset of auditory stimuli^33–37^. Others have suggested that spontaneous oscillations and the mid-latency response are related activities, as the amplitude of spontaneous 40 Hz gamma oscillations prior to the onset of AM tones at 40 Hz is inversely related to the magnitude of the subsequent middle latency response^36^.

Regardless, the larger ASSR to 40 Hz AM is thought to arise in part from the natural or preferred timing of local cell activity at that frequency, and numerous studies have linked the best AM frequency to the time course and magnitude of cell recruitment. Of note, the best AM frequency differs across subcortical and cortical recording locations^38^, reflecting varying degrees of cell recruitment^39,40^, variations in the timing of cell responses in different brain regions^38^, and changes in auditory responses across developmental stages^41–44^. It is thus possible that the ASSR changes across AM frequencies because the ASSR captures a summation of responses from different populations of cells, each with differing intrinsic properties and tendencies for transient versus oscillatory responses.

**Figure 1:**
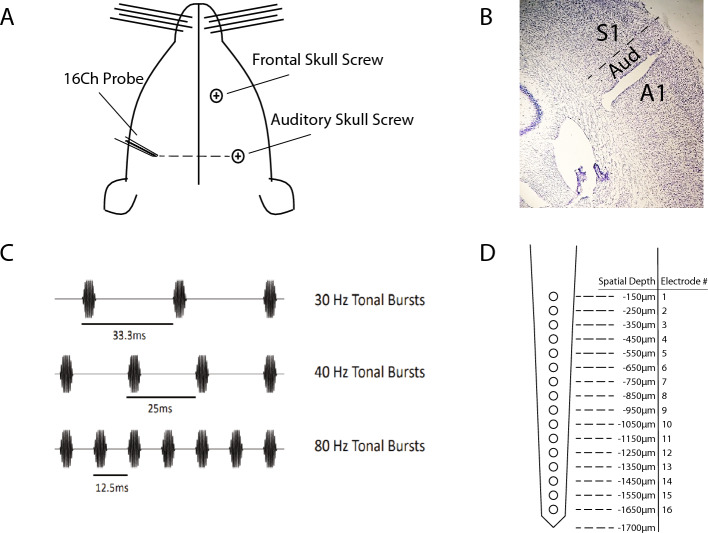
Auditory stimulus administration and the auditory steady state response (ASSR). (**A**) Illustration of the approximate recording locations for the experiment. (**B**) Example histology of probe location in the rat primary auditory cortex, near the boundary of dorsal secondary auditory cortex (S1 = primary somatosensory cortex, AUD = secondary auditory cortex, A1 = primary auditory cortex). (**C**) Example of the tonal bursts used to elicit ASSRs. (**D**) Illustration of the 16-channel probe used to record local field potential (LFP) activity, with corresponding estimates of cortical depths for each electrode.

**Figure 2:**
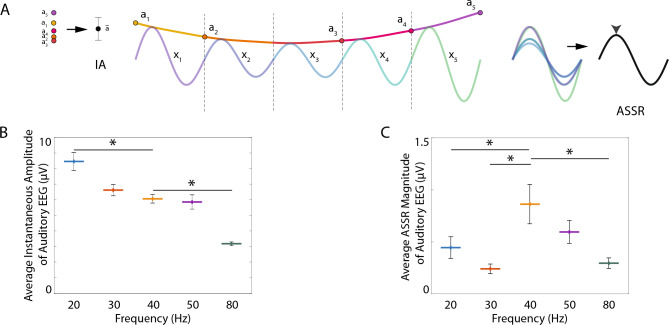
A resonant ASSR response to 40 Hz AM in signals acquired from the auditory EEG. (**A**) Illustration demonstrating the calculation of the average instantaneous amplitude (IA, left, black dot indicates the average of *a*_1_ through *a*_5_) and the ASSR (right, black waveform indicates the average of *x*_1_ through *x*_5_, and black arrow indicates the peak of the average waveform), implemented for calculation of (**B,C**), respectively. (**B**) Average IA of the filtered auditory EEG signal (bandpass filtered ± 1 Hz surrounding the AM frequency) for each AM frequency condition, averaged across the entire 15-minute duration of the stimulus condition. Thick lines indicate mean values, and the height of black lines indicate 95% confidence intervals. Asterisks indicate significant differences in pairwise comparisons to the 40 Hz AM condition (p < 0.05). (**C**) Modulation rate transfer function depicting the average ASSR across AM frequency conditions. Thick lines indicate mean values, and the height of black lines indicate 95% confidence intervals. Asterisks indicate significant differences in pairwise comparisons to the 40 Hz AM condition (p < 0.05).

**Figure 3:**
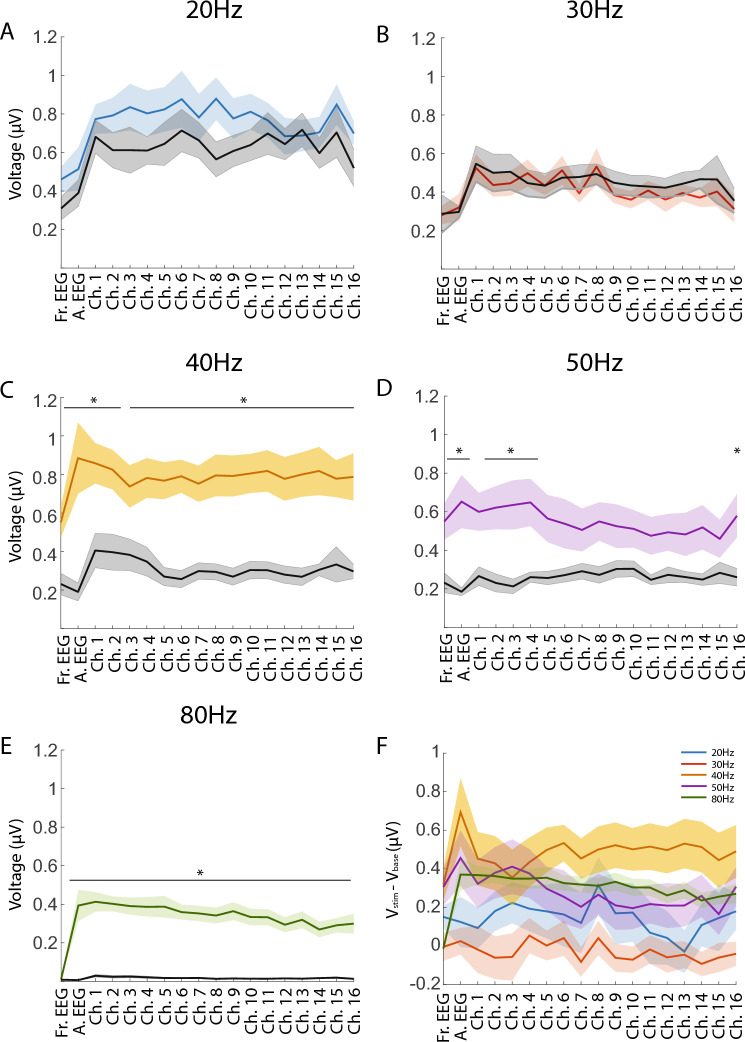
The ASSR during baseline (black) and sub-sampled auditory stimulus (colored) intervals. Each point on a line indicates the average ASSR across recordings sessions, calculated separately for each recording electrode. Shaded areas surrounding each line indicate the standard error of the mean. Each plot corresponds to a different AM frequency condition: (**A**) 20 Hz (blue), (**B**) 30 Hz (red), (**C**) 40 Hz (yellow), (**D**) 50 Hz (purple), and (**E**) 80 Hz (green). Asterisks and horizontal black bars in each plot indicate the electrode locations with responses significantly different from baseline responses (p < 0.01). (**F**) Mean differences between auditory stimulus and baseline responses, shown for each stimulation condition and each physical recording location. Colors are consistent with those in (**A–E**).

The present study aims to provide insight into the temporal coordination of activity within primary auditory cortex underlying resonant responses in the EEG. The ASSR is observable in multiple species (including humans^15,45–47^, non-human primates^48,49^, harbour porpoises^50^, rats^41,43,51,52^, mice^53,54^, cats^55^, bats^44^, rabbits^38,56^, Mongolian gerbils^39,57^, and chinchillas^58^) and can be recorded using multiple methods^38,52,59–61^. Accordingly, we conducted simultaneous silicon probe and skull EEG recordings in rats as they listened to tones modulated in amplitude at frequencies of 20, 30, 40, 50, and 80 Hz (Fig. 1). From a skull screw placed over the rat primary auditory cortex (henceforth termed auditory EEG), we observed the largest ASSR for 40 Hz AM, replicating prior studies^15,62^. To investigate the cause, we asked first whether 40 Hz neuronal oscillations are larger in signals acquired from the auditory EEG. Finding that the instantaneous amplitude of the AM frequency is in fact largest during 20 Hz AM (Fig. 2B), we then compared the magnitude of the ASSR to baseline activity recorded in the absence of periodic sound stimuli. Whereas the magnitude of the ASSR elicited by 20 and 30 Hz AM did not significantly differ from that recorded during the baseline period, 40 Hz AM and above substantially enhanced the ASSR (Fig. 3).

**Figure 4:**
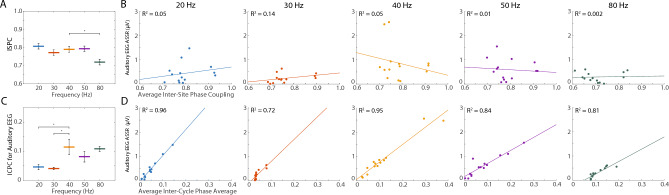
Relationship between the ASSR calculated from the auditory EEG, inter-site phase coupling (ISPC), and inter-cycle phase coupling (ICPC). (**A**) Average ISPC values during each AM frequency condition. Thick lines indicate mean values, and the height of black lines indicate 95% confidence intervals. Asterisks indicate significant differences in pairwise comparisons to the 40 Hz AM condition (p < 0.05). (**B**) The ASSR from the auditory EEG as a function of the ISPC values, shown separately for each AM frequency condition. Each dot corresponds to a different recording session, and each panel indicates a different AM frequency condition. Corresponding *R*^2^ values for the lines of best fit are shown in the upper left. (**C**) Average ICPC values during each AM frequency condition, for signals acquired from the auditory EEG. Thick lines indicate mean values, and the height of black lines indicate 95% confidence intervals. Asterisks indicate significant differences in pairwise comparisons to the 40 Hz AM condition (p < 0.05). (**D**) ASSR from the auditory EEG as a function of ICPC values, shown separately for each AM frequency condition. Each dot corresponds to a different recording session, and each panel indicates a different AM frequency condition. Corresponding *R*^2^ values for the lines of best fit are shown in the upper left.

**Figure 5:**
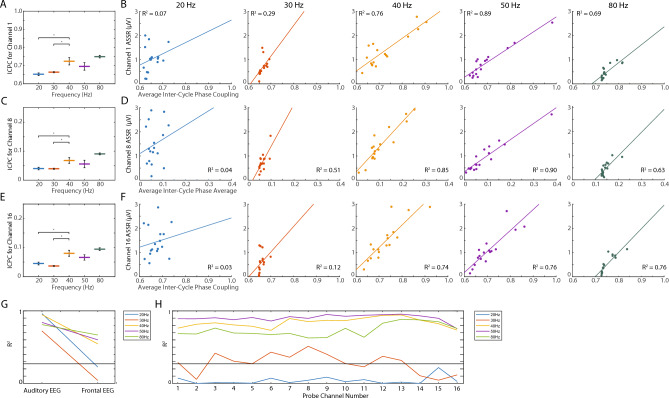
Relationship between the ASSR and ICPC for linear probe channels. (**A**) Average ICPC values during each AM frequency condition, for signals acquired from the most superficial channel (channel 1) on the linear probe. Thick lines indicate mean values, and the height of black lines indicate 95% confidence intervals. Asterisks indicate significant differences in pairwise comparisons to the 40 Hz AM condition (p < 0.05). (**B**) ASSR from the channel 1 electrode as a function of the ICPC values, shown separately for each AM frequency condition. Each dot corresponds to a different recording session, and each panel indicates a different AM frequency condition. Corresponding *R*^2^ values for the lines of best fit are shown in the upper left. (**C,E**) Same as in (**A**), for channels 8 and 16 of the linear probe, respectively. (**D,F**) Same as in (**B**), for channel 8 and 16 of the linear probe, respectively. (**G**) *R*^2^ for linear models describing relationships between ICPC values and the ASSR at each EEG recording location. Different color lines correspond to the different AM frequency conditions, with a black bar indicating the threshold for model significance (p < 0.05). (**H**) Same as in (**G**) for linear probe locations indicated along the x-axis.

**Figure 6:**
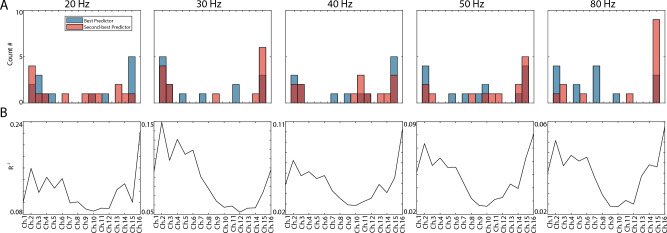
Modeling results assessing filtered probe signal (filtered ± 1 Hz surrounding the amplitude modulation frequency) covariance with the filtered auditory EEG signal. (**A**) Histogram indicating the spatial location of the channels that were the most predictive (blue) and the second most predictive (red) of the filtered auditory EEG. (**B**) Example *R*^2^ values for separate single LFP channel models used to predict the filtered auditory EEG signal.

Because EEG amplitude is sensitive to synchronous activity in cortical patches^63^, we then asked whether the ASSR was sensitive to the similarity of activity across A1 layers as reflected in the inter-site phase coherence of the LFP probes (Fig. 4A,B). Alternatively, the ASSR might be more related to the consistent timing of responses to each AM tone, as reflected in the inter-cycle phase coherence (Figs. 4C,D, 5). We demonstrate that temporal consistency of the response (that is, decreased latency variability) is strongly related to the magnitude of the ASSR in the EEG signal (Fig. 4), and that this relationship is more prominent at higher frequencies in the cortical LFP (Fig. 5). Finally, we model the auditory EEG signal from the cortical LFP activity, finding evidence for both superficial and deep generators (Fig. 6), and providing new insight into deep layer coordinators of the response.

## Results

### Resonance of the ASSR

We constructed a modulation rate transfer function to depict changes in the ASSR across different frequencies of amplitude modulation (AM) (Fig. 2C). We first calculated the ASSR from skull screw electrodes placed over the rat primary auditory cortex (termed the auditory EEG) or frontal cortex (termed the frontal EEG). The ASSR changed dramatically across AM frequencies (Table S1). A maximum was observed for 40 Hz AM, and this was true for both the auditory (Fig. 2C) and frontal (Fig. S1) EEG. Individual pairwise comparisons with the 40 Hz AM condition revealed that 40 Hz AM elicited a significantly larger ASSR in the auditory EEG than 20, 30, and 80 Hz AM (Table S1).

This result is in contrast to a strikingly different trend in the average instantaneous amplitude (IA) observed from the auditory EEG electrode across AM frequencies, which decreased as the AM frequency increased (Fig. 2B, Fig. S2). Individual pairwise comparisons with the 40 Hz AM condition revealed that 40 Hz oscillations during 40 Hz AM were substantially smaller than 20 Hz and larger than 80 Hz oscillations during their respective conditions (Tukey’s Honestly Significant Difference Test, p < 0.05, Fig. 2B).

To visually characterize the ASSR observed from the auditory EEG across AM frequencies, single cycle (Fig. S3) and 1s (Fig. S4) averages were calculated. A consistent response to each AM frequency could be observed in this manner, and this response appeared to be paced by the AM frequency. Power spectral density estimates of the averaged 1s intervals revealed frequency components within the averaged signal (Fig. S4C,D), with the results largely mimicking the results observed in our modulation rate transfer function depiction of the ASSR (Fig. 2C). In particular, the strongest pacing by the auditory stimulus was observed during 40 Hz AM.

We next constructed modulation rate transfer functions for signals acquired from intracranial probe electrodes. The ASSR varied significantly across AM frequencies for every probe electrode location (Table S1). Individual pairwise comparisons with the 40 Hz AM condition revealed that the ASSR was significantly larger during 40 Hz AM than during 30 Hz or 80 Hz AM for all probe locations (Tukey’s Honestly Significant Difference Test, p < 0.05, Table S1). The ASSR for 50 Hz AM was significantly smaller than the ASSR for 40 Hz AM only for deep probe channels, and no intracranial probe locations showed any significant differences between the ASSR observed during 20 and 40 Hz AM (Tukey’s Honestly Significant Difference Test, p < 0.05, Table S1).

To reveal the extent that the ASSR during auditory stimulus conditions differed from the ASSR observed during a baseline interval at the end of recording sessions, 15-min auditory stimulus intervals were subsampled to match the duration of 5-min baseline intervals. Magnitude comparisons indicated there were no significant increases in the ASSR from baseline during the 20 or 30 Hz AM conditions for any electrode (Fig. 3A,B, Table S2). During the 40 Hz AM condition, all EEG and probe channels except for channel 3 exhibited significant increases in the ASSR from baseline (Fig. 3C, Table S2). During the 50 Hz AM condition, the frontal EEG, the auditory EEG, and select superficial and deep electrodes exhibited significant increases in the ASSR from baseline (Fig. 3D, Table S2). Lastly during the 80 Hz AM condition, the auditory EEG and all probe channels exhibited significant increases in the ASSR from baseline (Fig. 3E, Table S2). In contrast, only the auditory and frontal EEG electrodes exhibited significant increases in the instantaneous amplitude (IA) of oscillations compared to baseline. Specifically, the auditory EEG had significant increases in the IA of 20 Hz, 50 Hz, and 80 Hz oscillations during stimulus epochs compared to baseline (Fig. S5). The frontal EEG had significant IA increases for only 80 Hz oscillations during stimulus epochs compared to baseline (Fig. S5).

### No significant influence of cross-layer synchrony on EEG response

A significant difference in inter-site phase coupling (ISPC) was observed across AM frequencies (repeated measures ANOVA, d.f. = 4, F = 48.375, p < 1.0 × 10$^{-9}$), largely reflecting reduced ISPC values in the 80 Hz AM condition (Fig. 4A). Individual pairwise comparisons with the 40 Hz AM condition revealed that there were no significant differences between ISPC values during the 40 Hz AM condition and ISPC values during 20 Hz, 30 Hz, or 50 Hz AM conditions (Tukey’s Honestly Significant Difference test, p > 0.05). Notably, ISPC values during 80 Hz AM were significantly smaller than ISPC values during 40 Hz AM (Tukey’s Honestly Significant Difference test, p < 0.05). To determine how ISPC values might covary with the ASSR calculated from the auditory EEG, a linear model was fit between these two measures for each recording session. R$^{2}$ values suggest there were no significant relationships between the two values during any of the auditory stimulus conditions (t-statistic, p > 0.05, scatter and R^2^ values for auditory EEG in Fig. 4B, scatter and R^2^ values for the frontal EEG in Fig. S6).

### ASSR covaries with increased cycle phase consistency at high frequencies

The inter-cycle phase coupling (ICPC) changed significantly across AM frequencies for all EEG and probe electrodes (Fig. 4C, Table S3). Individual pairwise comparisons with the 40 Hz AM condition revealed that ICPC values for all recording locations were significantly higher during 40 Hz AM than during 20 or 30 Hz AM (Table S3). For select deep electrode channels, ICPC values during 40 Hz AM were significantly lower than ICPC values during 80 Hz AM (Table S3). For channel 15 only, ICPC values during 40 Hz AM were also significantly higher than ICPC values observed during 50 Hz AM (Table S3). To determine how ICPC values might covary with the ASSR calculated from the auditory EEG, a linear model was fit between these two measures for each recording session. For all AM frequencies, the ASSR calculated from the auditory EEG significantly covaried with ICPC values (Figs. 4D, 5G, Table S4). For the 40, 50, and 80 Hz AM conditions, the ASSR calculated from all electrodes significantly covaried with ICPC values (t-statistic, p < 0.05, Fig. 5B,D,F,H). Of note, for the 20 Hz AM condition, only the ASSR calculated from the auditory EEG significantly covaried with ICPC values (p < 0.05, Figs. 4D, 5B,D,F,H). For the 30 Hz AM condition, ICPC values significantly covaried with the ASSR for the auditory EEG and a subset of probe electrodes (electrodes 3, 6, 7, 8, 9, 10, 12, and 13) (p < 0.05, Fig. 5H).

### Modeling EEG signals from the LFP

We constructed linear models that use single LFPs to describe relationships between filtered LFP (filtered ± 1 Hz surrounding the amplitude modulation frequency of interest) and EEG signals. These models revealed that the most predictive channels were often the most superficial and deep channels (Fig. 6A). The second most predictive channel was not always the channel farthest from the most predictive channel, for either the auditory or frontal EEG (example shown in Fig. 6B). For the frontal EEG, the most predictive channels tended to be the deepest channels (Fig. S7).

## Discussion

Our study probed the neurobiological underpinnings of the auditory steady-state response (ASSR), with a particular focus on the differential recruitment of networks within primary auditory cortex (A1) across changing modulation frequencies. We delivered blocks of tones modulated in amplitude at 20, 30, 40, 50, and 80 Hz, and confirmed that the largest ASSR was observed during the 40 Hz modulation rate condition (Fig. 2C). Critically, responses during 40 Hz AM were not larger in instantaneous amplitude (Fig. 2B, Fig. S5), but rather exhibited less latency variability than those elicited by stimuli at the other amplitude-modulation (AM) frequencies (Fig. 4C). Further, the size of the ASSR showed no relationship to inter-site phase coupling as might be expected if the large ASSR to 40 Hz AM was due to phase alignment across cortical layers (Fig. 4A,B). This suggests the ASSR reflects the recruitment of multiple oscillators across A1 that are optimally or more consistently engaged at 40 Hz AM, but not uniformly engaged at the same time. Results thus reveal that the size of the ASSR is strongly related to the temporal consistency of cortical network recruitment, and that such consistency depends on the modulation frequency.

Although it is well established that the size of the ASSR in EEG recordings is closely related to the temporal consistency of the response^54,62,64–66^, its relationship to the amplitude of endogenous oscillations has been less clear. The present study allowed us to decouple the impact of these two factors on the ASSR in the EEG recordings because 20 Hz AM elicited the largest instantaneous amplitude response (Fig. 2C), while exhibiting relatively low temporal consistency (Fig. 4C), and 40 Hz AM elicited a response with a relatively small instantaneous amplitude (Fig. 2B), while exhibiting the greatest level of temporal consistency. The largest ASSR calculated from the auditory EEG was elicited not by 20 Hz AM, but by 40 Hz AM (Fig. 2C). Indeed, 40 Hz AM tones had no impact on the average instantaneous amplitude of 40 Hz oscillations (Fig. S5), even as they elicited a large increase in the ASSR in the auditory EEG (Fig. 3). Of note, there is some evidence to suggest that recruitment into endogenous oscillations is incompatible with recruitment by the envelope of an auditory stimulus^36,67^, though both mechanisms may leverage the input preferences of neurons in local circuits (e.g., for specific modulation frequencies), or the natural timing of their responses. Our results highlight the fact that the mere presence of oscillatory activity at the modulation frequency need not imply that neuronal oscillations are consistently aligned with the stimulus envelope.

Critically, the relationship between temporal consistency and the ASSR measured in the auditory EEG is not ubiquitous, as it is most relevant for AM frequencies above 30 Hz. In the present study, the linear relationship between inter-cycle phase coherence (ICPC) and the ASSR was present at all EEG and LFP probe signals for 40, 50, and 80 Hz AM. In contrast, for 30 Hz AM, this relationship held only at a few, select electrodes, and for 20 Hz AM, it was restricted to the auditory EEG (Fig. 5). This discrepancy, along with the relatively low ICPC levels in the LFP elicited by lower frequency AM, suggest differences in the nature of auditory network recruitment for sounds at AM frequencies at or below 30 Hz versus those higher in the gamma band (traditionally defined in the range of 30–150 Hz).

The frequency-dependent nature of the ASSR to periodic stimuli may be driven in part by the frequency-dependent engagement of specific neural generators. The AM frequencies used here would likely recruit activity from multiple generators, including prominent contributions from local cortical generators at frequencies below 50 Hz and the recruitment of subcortical generators at higher frequencies^38,41,68–71^. Consequently, the troughs of our modulation rate transfer functions (e.g. 30 Hz), may arise from a spatial misalignment of currents from these various ASSR generators^38^. However, the decreased temporal consistency of the response to 30 Hz AM (indexed by the low ICPC values reported here) suggests that the phase consistency of the generators is also more variable, perhaps due to inconsistent or uncoordinated recruitment of the generators. Likewise, the 40 Hz peak in the modulation transfer function may reflect greater synchrony among recruited populations.

This in turn raises the question of what leads to the enhanced temporal consistency of the neural responses underlying the large ASSR to 40 Hz AM. One salient possibility is that subpopulations of neurons in A1 exhibit resonance at 40 Hz, contributing to the generation of relatively larger scale, consistent oscillations given inputs near that frequency^72^. This would also lead to greater synchrony among recruited populations, which has been reported in previous studies at the best AM frequency^38^. In subcortical recordings of the ASSR, it has also been shown that the best AM frequency is tightly linked to the input preferences of recruited neurons and the time course of their responses^38,73^, with the best AM frequency often evolving over the course of development as the intrinsic properties of these neurons change^41–44^.

Taken together, this suggests a strong relationship between enhanced temporal consistency of responses for certain AM frequencies and the resonance profiles of recruited subpopulations. It is also possible that interactions between neurons in A1 have a stereotyped or optimal time course that is best paced by 40 Hz stimuli. In either case, the intrinsic properties of participating neurons give rise to ion currents and resulting fluctuations in the LFP with stereotyped timing^74^. Optimal pacing of the auditory network at 40 Hz may thus arise because this frequency is complementary to the time course of natural ionic fluctuations and interactions.

Our findings thus provide insight into potential underlying neural differences in patient populations with atypical ASSRs in EEG recordings^2–11^. Previous investigators have speculated that the ability to recruit auditory networks in gamma frequency activity is altered in these populations^75^, resulting in a lower amplitude ASSR^76–79^. While it may indeed be the case that low gamma oscillatory power contributes to a reduced ASSR to 40 Hz AM in these groups, here we find the temporal coordination of the 40 Hz AM response has a far greater impact on the ASSR than the ability to generate large 40 Hz oscillations. These results imply that deficits in patient groups may stem from intrinsic cellular or circuit changes that modify endogenous stereotyped network timing in this frequency range. Such changes could influence both the ability to generate gamma oscillations and the temporal consistency of responses to AM stimuli in the gamma frequency.

Gamma oscillations are often thought to be generated through interactions between excitatory and inhibitory neurons, with the frequency of the rhythm dictated by the time course of recovery from inhibition^80,81^. As A1 contains several inhibitory interneuron subtypes, spread across all cortical layers^82^, results of the present study align with the suggestion that interneuronal recruitment contributes to both the generation of endogenous gamma oscillations in A1 and the ASSR to 40 Hz AM^76–79^. For example, parvalbumin positive interneurons in layer VI send inhibitory projections across all cortical layers^83^, allowing them to impose temporally coordinated inhibitory constraints. Neuronal populations in layers V/VI, with their prominent projections to layer IV and thalamus, may play a key role in the recruitment and maintenance of the ASSR and the high temporal consistency of the response.

Accordingly, results here indicate that ion currents in deep (V/VI) layers of A1 are tightly correlated with the auditory EEG signal. These layers contain a diversity of neuron types with both thalamic and cortical targets^84–86^. Further, the neurons in the deep layers of A1 are in a prime anatomical position to exert a powerful coordinating influence on the rest of the network. A previous study noted that the ASSR is largest in the granular layer (layer IV)^54^, suggesting strong coordinating inputs in the region. Approximately half of the input to layer IV originates from within cortex, with prominent projections from layer VI^87,88^. Projections from layer VI also differ from thalamic projections to layer IV in that they uniquely activate metabotropic receptors and facilitate EPSPs in response to repetitive stimuli^89,90^.

Previous studies examining the cellular contributions to the ASSR and 40 Hz gamma oscillations have focused on the role of NMDA and GABA receptors. Blockade of either receptor type influences the amplitude of the ASSR to 40 Hz AM, with NMDA receptor blockade dynamically decreasing or enhancing the ASSR to 40 Hz AM over time^64,65,91^. Several previous studies have also implicated NMDA and GABAergic receptors in the generation of 40 Hz oscillations in auditory networks^76–79,91^. These studies hypothesize that interactions between excitatory and inhibitory neurons, or NMDA receptor regulation of GABAergic neuron activity support intrinsic network gamma oscillations and thus the temporal consistency of responses in that range.

Lastly, our study provides further insight into a long-standing debate regarding the contributions of phasic middle latency responses or endogenous oscillations to the ASSR. This debate is a part of a larger controversy regarding the extent that steady-state responses reflect event-related responses that are paced by external stimuli or the recruitment of oscillating networks of neurons, a distinction that bears on what these responses reveal about the underlying network. The results of our study suggest that both possibilities may be true at different frequencies. At lower frequencies (30 Hz and below), the network recruits more variable responses to individual stimuli in line with an event-related response. In contrast, AM sounds at higher frequencies (40 Hz and above) elicit a more consistent and stereotyped response resembling an oscillation, with the enhanced consistency at particular frequencies (40 Hz) resembling neural resonance. Moreover, recruitment of local cell populations through either mechanism could vary across different AM frequencies, resulting in the activation of distinct subpopulations^38–40^. Overall, results suggest auditory network recruitment by repetitive stimuli occurs in multiple forms, marked by sharp changes in the temporal consistency of the response as the frequencies pacing the network change. Our study reveals that this frequency-dependent shift in auditory network recruitment substantially contributes to the unique network state underlying the ASSR to 40 Hz AM.

## Methods

### Rats

All animal procedures were performed in accordance with University of California, San Diego Institutional Animal Care and Use Committee (IACUC) and Association for Assessment and Accreditation of Laboratory Animal Care International (AAALAC) guidelines. The experimental protocol was approved by the University of California, San Diego Institutional Animal Care and Use Committee (IACUC, protocol S16215). Subjects were three male and three female Long-Evans rats (Charles River Laboratories), between 6 and 18 months old. Rats were housed individually or with a single other rat, and maintained on a 12-h light/dark cycle. All neural recordings were performed during the light cycle. Rats received food and water ad libitum. At the end of the experiment, rats were euthanized with a pentobarbital sodium and phenytoin sodium solution (Euthasol, Virbac Corporation, Texas). The authors have complied with ARRIVE guidelines.

### Surgery and histology

Each rat was surgically implanted with a 16-channel single shank silicon probe (NeuroNexus Technologies, Ann Arbor, MI; Qualia Labs, Dallas, TX). Probe shanks were 5 mm in length, and electrodes were spaced 100 $\upmu$m apart along the lowest 1550 $\upmu$m of the shank. A diagram outlining the electrode configuration of the silicon probes is shown in Fig. 1D. Probes were implanted in left primary auditory cortex near the boundary of dorsal secondary auditory cortex (A/P = − 4.0 mm; M/L = − 7.2 mm), and the probe was placed 1.65 mm into the brain to target all layers of primary auditory cortex (Fig. 1A,B). To record skull EEG, a skull screw was placed above right primary auditory cortex (A/P = − 4.0 mm; M/L = + 7.2 mm, termed auditory EEG). In four of the rats, an additional skull screw was placed above right frontal association cortex (A/P = 5.2 mm; M/L = − 2.0 mm, termed frontal EEG). A skull screw placed over left frontal association cortex served as a ground, while an additional skull screw placed over cerebellum served as a reference. Probe locations were visualized via a Nissl stain in 40 $\upmu$m coronal sections (Fig. 1B).

### Neural recordings

Signals were amplified by a preamplifier 20$\times$ and amplified again to 4000–6000$\times$ (Plexon, Dallas, TX). Local field potential (LFP) and skull electroencephalography (EEG) signals were digitally isolated with a band-pass filter from 1 to 500 Hz and notch filtered to remove 60 Hz electrical noise. LFP and EEG channels were globally referenced to a skull screw above the cerebellum. Movement artifacts were first targeted by isolating time points where the signal amplitude was more than 2.5$\times$ the average amplitude per recording session, and then were manually reviewed for further isolation. Five of the six rats underwent three recording sessions, and one rat underwent two recording sessions.

### Auditory steady state stimulus

Auditory stimuli were amplitude-modulated (AM) tones with a 100 kHz sampling frequency, 2 kHz carrier frequency, and 5ms duration. This carrier frequency was chosen due to the fact that it is detectable by both rats and humans at a wide range of ages when presented at 80dB SPL, allowing for greater translatability of results^92,93^. Each tone was amplitude modulated with a trapezoidal wave transposed onto them. Amplitude-modulated tones had a 25% rise and fall time (of the 5 ms duration), ensuring a short onset and offset of the stimuli to avoid auditory chirps. Each trial block consisted of a continuous 15 min of AM tones, presented at 20, 30, 40, 50, or 80 Hz. The order of blocks was randomized for each recording session. A schematic depicting example auditory stimuli is shown in Fig. 1C. Between each 15 min block, an interval without any auditory stimuli (silence) of at least one minute in duration was implemented to limit any residual effects generated from stimuli presented during the previous block. Approximately 65–90% of trials within each block remained after the removal of mechanical artifact (as described above), resulting in > 11,000 trials per block. During auditory stimulus presentation, rat subjects were placed into an enclosed environment with speakers placed directly outside the environment. Rats were free to roam around in their enclosed environment during recording sessions. Before each recording session, the volume of the stimulus inside the environment was verified to be at 80dB SPL. Following the delivery of auditory stimuli, a 5-min. baseline was recorded at the end of each recording session.

### Auditory steady state response

To quantify voltage responses elicited by the auditory stimulus, the LFP and EEG signals were first bandpass filtered using a 3rd order Butterworth filter in a narrow (± 1 Hz) range around the AM frequency of interest (e.g. for 20 Hz, the data was bandpass filtered from 19 to 21 Hz). The 15-min trials were then binned into one-cycle segments of the auditory stimulus and averaged across bins. The ASSR for a given amplitude-modulation (AM) frequency was then calculated as the maximum voltage of the resulting averaged waveform. Since the averaged waveform is approximately a sinusoidal shape, the maximum value is similar to calculating the distance from the mean (0mV) of the averaged waveform. A schematic depicting calculation of the ASSR is shown in Fig. 2A (right). In addition, we assessed the average instantaneous amplitude (IA) by first calculating the absolute value of the complex Hilbert transform of the filtered signal for the entire 15-min interval. We then identified the peak value of the instantaneous amplitude for each trial (for each AM tone), and then averaged the peak values (Fig. 2A, left). The ASSR and IA were also calculated using less narrow filters of the LFP and EEG signals (Fig. S2, for ± 5 Hz the AM frequency, 10–90 Hz, or no filter). When comparing ASSR and IA values across baseline and auditory stimulus blocks, the 15-min auditory stimulus blocks were subsampled to match the duration of the 5-min baseline intervals. Of note, given higher noise levels or variability in baseline signals, the length of baseline recordings may limit the identification of robust but modest changes during auditory stimulus blocks.

### Current source density analysis

To identify current sources and sinks resulting from the auditory stimulus, we calculated the second order spatial derivative of narrowly filtered probe LFPs. For every spatial location of a probe electrode *s* at time *t* the current source density of the signal was calculated as:

1 $$\begin{aligned} CSD_{s,t} = \frac{\frac{LFP_{s+1,t} + LFP_{s+2,t}}{2}+\frac{LFP_{s-1,t}+LFP_{s-2,t}}{2} - 2LFP_{s,t}}{z^{2}}, \end{aligned}$$

where z represents the spacing between probe electrodes (0.1 mm).

### Inter-site phase coupling

To determine the phase alignment of responses across probe recording locations, the inter-site phase coupling (ISPC) was calculated as:

2 $$\begin{aligned} ISPC_{t} = \bigg|\frac{\sum _{l = 1}^{16}e^{i\psi _{l,t}}}{16}\bigg|, \end{aligned}$$

where *l* represents the spatial location of each electrode of the probe, and $\psi$ is the phase of a response, determined by narrowly filtering (± 1 Hz) the local field potential signal around an amplitude modulation frequency of interest and calculating the arctangent of the Hilbert transform. The ISPC value characterizes the phase consistency of responses across all probe electrodes. ISPC values are between 0 and 1, with 0 indicating no phase consistency across all probe electrodes, and 1 indicating perfect phase consistency across all probe electrodes. To assess the overall influence of a particular auditory stimulus upon the phase consistency of responses across electrodes, ISPC values were averaged for the duration of a stimulus condition, or 15-min trial.

### Inter-cycle phase coupling

To characterize the temporal consistency of the responses acquired from a single electrode, the inter-cycle phase coupling (ICPC) was calculated as:

3 $$\begin{aligned} ICPC_{\tau } = \bigg|\frac{\sum _{c = 1}^{m}e^{i\psi _{c,\tau }}}{m}\bigg|, \end{aligned}$$

where $\tau$ is a single time point within the period of a single auditory stimulus cycle, *m* is the number of cycles, and $\psi$ is is the phase of a response, determined by narrowly filtering (± 1 Hz) the local field potential signal around an AM frequency of interest and calculating the arctangent of the Hilbert transform. The ICPC value characterizes the phase consistency of the responses across presentation cycles. ICPC can take on values between 0 and 1, with 0 indicating no phase consistency across cycles, and 1 indicating perfect phase consistency across trials. To assess the overall influence of a particular AM frequency upon the phase consistency of responses on an electrode, ICPC values were calculated for the duration of a 15-min trial. ICPC values were obtained for the different time points within an auditory stimulus cycle, and then averaged.

### Assessing phase consistency covariates of the ASSR

We performed simple linear regression to assess relationships between the ASSR and the spatial and temporal consistency of responses. To determine whether the phase consistency of responses across probe electrodes related to the ASSR acquired from EEG electrodes, linear models were constructed to relate ISPC values to the ASSR. To determine whether the temporal consistency of responses on a single electrode influenced the ASSR on the same electrode, linear models were constructed to relate average individual electrode ICPC values to the ASSR on the same electrode. These latter models were constructed for each electrode (16 probe electrodes, one frontal EEG electrode, and one auditory EEG electrode) and AM frequency condition separately. Model goodness of fit was assessed via the coefficient of determination ($R^2$).

### Modeling EEG signals from the LFP

To evaluate the extent that individual LFPs covaried with the EEG signals, and to what extent the signal from each probe channel contains unique information about the variance of the EEG signals, linear models relating individual LFPs with EEG signals were constructed. These models used the filtered LFP (narrowly filtered ± 1 Hz around a stimulus frequency of interest) from a single probe channel as model regressors. $R^2$ values were used to compare model effectiveness.

### Supplementary Information

Supplementary Information.

## Data Availability

Data reported in this paper will be shared by the corresponding author (lrangel@ucsd.edu) upon request. Any additional information required to reanalyze the data reported in this paper is also available from the corresponding author (lrangel@ucsd.edu) upon request.

## Code availability

This paper does not report original code.

## References

[1] Regan, D. Human brain electrophysiology. In *Evoked Potentials and Evoked Magnetic Fields in Science and Medicine* (1989).

[2] Osipova, D., Pekkonen, E. & Ahveninen, J. Enhanced magnetic auditory steady-state response in early Alzheimer’s disease. *Clin. Neurophysiol.* **117**, 1990–1995 (2006). 16887381 10.1016/j.clinph.2006.05.034

[3] Spencer, K. M., Salisbury, D. F., Shenton, M. E. & McCarley, R. W. -band auditory steady-state responses are impaired in first episode psychosis. *Biol. Psychiatry* **64**, 369–375 (2008). 18400208 10.1016/j.biopsych.2008.02.021 PMC2579257

[4] Rass, O. *et al.* Auditory steady state response in bipolar disorder: Relation to clinical state, cognitive performance, medication status, and substance disorders. *Bipolar Disord.* **12**, 793–803 (2010). 21176026 10.1111/j.1399-5618.2010.00871.x PMC3060563

[5] O’Donnell, B. F. *et al.* The auditory steady-state response (ASSR): A translational biomarker for schizophrenia. *Suppl. Clin. Neurophysiol.* **62**, 101–112 (2013). 24053034 10.1016/B978-0-7020-5307-8.00006-5 PMC4959266

[6] Thuné, H., Recasens, M. & Uhlhaas, P. J. The 40-Hz auditory steady-state response in patients with schizophrenia: A meta-analysis. *JAMA Psychiatry* **73**, 1145–1153 (2016). 27732692 10.1001/jamapsychiatry.2016.2619

[7] Isomura, S. *et al.* Differentiation between major depressive disorder and bipolar disorder by auditory steady-state responses. *J. Affect. Disord.* **190**, 800–806 (2016). 26625092 10.1016/j.jad.2015.11.034

[8] Khaleghi, A., Zarafshan, H. & Mohammadi, M. R. Visual and auditory steady-state responses in attention-deficit/hyperactivity disorder. *Eur. Arch. Psychiatry Clin. Neurosci.* **269**, 645–655 (2019). 29789937 10.1007/s00406-018-0902-6

[9] Oda, Y. *et al.* Gamma band neural synchronization deficits for auditory steady state responses in bipolar disorder patients. *PLoS ONE* **7**, e39955 (2012). 22792199 10.1371/journal.pone.0039955 PMC3390322

[10] Seymour, R. A., Rippon, G., Gooding-Williams, G., Sowman, P. F. & Kessler, K. Reduced auditory steady state responses in autism spectrum disorder. *Mol. Autism* **11**, 1–13 (2020). 32611372 10.1186/s13229-020-00357-y PMC7329477

[11] Sugiyama, S. *et al.* The auditory steady-state response: Electrophysiological index for sensory processing dysfunction in psychiatric disorders. *Front. Psychol.* **12**, 644541 (2021). 10.3389/fpsyt.2021.644541 PMC7991095 33776820

[12] Baltus, A. & Herrmann, C. S. Auditory temporal resolution is linked to resonance frequency of the auditory cortex. *Int. J. Psychophysiol.* **98**, 1–7 (2015). 26268810 10.1016/j.ijpsycho.2015.08.003

[13] Zaehle, T., Lenz, D., Ohl, F. & Herrmann, C. Resonance phenomena in the human auditory cortex: Individual resonance frequencies of the cerebral cortex determine electrophysiological responses. *Exp. Brain Res.* **203**, 629–635 (2010). 20449728 10.1007/s00221-010-2265-8

[14] Baltus, A. & Herrmann, C. S. The importance of individual frequencies of endogenous brain oscillations for auditory cognition—A short review. *Brain Res.* **1640**, 243–250 (2016). 26453287 10.1016/j.brainres.2015.09.030

[15] Picton, T. W., John, M. S., Dimitrijevic, A. & Purcell, D. Human auditory steady-state responses: Respuestas auditivas de estado estable en humanos. *Int. J. Audiol.* **42**, 177–219 (2003). 12790346 10.3109/14992020309101316

[16] Hamm, J. P., Gilmore, C. S. & Clementz, B. A. Augmented gamma band auditory steady-state responses: Support for nmda hypofunction in schizophrenia. *Schizophr. Res.* **138**, 1–7 (2012). 22542616 10.1016/j.schres.2012.04.003 PMC3601795

[17] Kwon, J. S. *et al.* Gamma frequency-range abnormalities to auditory stimulation in schizophrenia. *Arch. Gen. Psychiatry* **56**, 1001–1005 (1999). 10565499 10.1001/archpsyc.56.11.1001 PMC2863027

[18] Brenner, C. A., Sporns, O., Lysaker, P. H. & O’Donnell, B. F. Eeg synchronization to modulated auditory tones in schizophrenia, schizoaffective disorder, and schizotypal personality disorder. *Am. J. Psychiatry* **160**, 2238–2240 (2003). 14638599 10.1176/appi.ajp.160.12.2238

[19] Light, G. A. *et al.* Gamma band oscillations reveal neural network cortical coherence dysfunction in schizophrenia patients. *Biol. Psychiatry* **60**, 1231–1240 (2006). 16893524 10.1016/j.biopsych.2006.03.055

[20] Griskova-Bulanova, I., Ruksenas, O., Dapsys, K., Maciulis, V. & Arnfred, S. M. Distraction task rather than focal attention modulates gamma activity associated with auditory steady-state responses (ASSRS). *Clin. Neurophysiol.* **122**, 1541–1548 (2011). 21377412 10.1016/j.clinph.2011.02.005

[21] Stapells, D. R., Galambos, R., Costello, J. A. & Makeig, S. Inconsistency of auditory middle latency and steady-state responses in infants. *Electroencephalogr. Clin. Neurophysiol. Evoked Potentials Sect.* **71**, 289–295 (1988). 10.1016/0168-5597(88)90029-9 2454794

[22] Galambos, R., Makeig, S. & Talmachoff, P. J. A 40-Hz auditory potential recorded from the human scalp. *Proc. Natl. Acad. Sci.* **78**, 2643–2647 (1981). 6941317 10.1073/pnas.78.4.2643 PMC319406

[23] Plourde, G., Stapells, D. R. & Picton, T. W. The human auditory steady-state evoked potentials. *Acta Otolaryngol.* **111**, 153–160 (1991). 1814147 10.3109/00016489109136793

[24] Bohórquez, J. & Özdamar, Ö. Generation of the 40-Hz auditory steady-state response (ASSR) explained using convolution. *Clin. Neurophysiol.* **119**, 2598–2607 (2008). 18818122 10.1016/j.clinph.2008.08.002

[25] Azzena, G. B. *et al.* Stimulus rate effects. Generation of human auditory steadystate responses (SSRS). i. *Hear. Res.* **83**, 1–8 (1995). 7607975 10.1016/0378-5955(94)00184-R

[26] Conti, G., Santarelli, R., Grassi, C., Ottaviani, F. & Azzena, G. B. Auditory steady-state responses to click trains from the rat temporal cortex. *Clin. Neurophysiol.* **110**, 62–70 (1999). 10348322 10.1016/S0168-5597(98)00045-8

[27] Galambos, R. Tactile and auditory stimuli repeated at high rates (30–50 per s) produce similar event related potentials. *Ann. N. Y. Acad. Sci.* **338**, 722–726 (1980). 10.1111/j.1749-6632.1980.tb19406.x 6953908

[28] Santarelli, R. *et al.* Addition of responses to individual stimuli. Generation of human auditory steady-state responses (SSRS). ii. *Hear. Res.* **83**, 9–18 (1995). 7607994 10.1016/0378-5955(94)00185-S

[29] Galambos, R. & Makeig, S. Dynamic changes in steady-state responses. In *Dynamics of Sensory and Cognitive Processing by the Brain* (eds Galambos, R. & Makeig, S.) 103–122 (Springer, 1988).

[30] Bressler, S. L. The gamma wave: A cortical information carrier? *Trends Neurosci.* **1**, 1 (1990). 10.1016/0166-2236(90)90039-d 1693231

[31] Barth, D. S. & MacDonald, K. D. Thalamic modulation of high-frequency oscillating potentials in auditory cortex. *Nature* **383**, 78–81 (1996). 8779725 10.1038/383078a0

[32] Brosch, M., Budinger, E. & Scheich, H. Stimulus-related gamma oscillations in primate auditory cortex. *J. Neurophysiol.* **87**, 2715–2725 (2002). 12037173 10.1152/jn.2002.87.6.2715

[33] Fukushima, M., Saunders, R. C., Leopold, D. A., Mishkin, M. & Averbeck, B. B. Spontaneous high-gamma band activity reflects functional organization of auditory cortex in the awake macaque. *Neuron* **74**, 899–910 (2012). 22681693 10.1016/j.neuron.2012.04.014 PMC3372858

[34] Brett, B. & Barth, D. S. Subcortical modulation of high-frequency (gamma band) oscillating potentials in auditory cortex. *J. Neurophysiol.* **78**, 573–581 (1997). 9307095 10.1152/jn.1997.78.2.573

[35] Lakatos, P. *et al.* An oscillatory hierarchy controlling neuronal excitability and stimulus processing in the auditory cortex. *J. Neurophysiol.* **94**, 1904–1911 (2005). 15901760 10.1152/jn.00263.2005

[36] Başar, E., Rosen, B., Başar-Eroglu, C. & Greitschus, F. The associations between 40 Hz-eeg and the middle latency response of the auditory evoked potential. *Int. J. Neurosci.* **33**, 103–117 (1987). 3610489 10.3109/00207458708985933

[37] Joliot, M., Ribary, U. & Llinas, R. Human oscillatory brain activity near 40 Hz coexists with cognitive temporal binding. *Proc. Natl. Acad. Sci.* **91**, 11748–11751 (1994). 7972135 10.1073/pnas.91.24.11748 PMC45309

[38] Kuwada, S. *et al.* Sources of the scalp-recorded amplitude-modulation following response. *J. Am. Acad. Audiol.* **13**, 188–204 (2002). 12025895 10.1055/s-0040-1715963

[39] Heil, P., Schulze, H. & Langner, G. Ontogenetic development of periodicity coding in the inferior colliculus of the Mongolian Gerbil. *Audio Neurosci.* **1**, 363–383 (1995).

[40] Walton, J. P., Simon, H. & Frisina, R. D. Age-related alterations in the neural coding of envelope periodicities. *J. Neurophysiol.* **88**, 565–578 (2002). 12163510 10.1152/jn.2002.88.2.565

[41] Prado-Gutierrez, P. *et al.* Maturational time course of the envelope following response to amplitude-modulated acoustic signals in rats. *Int. J. Audiol.* **51**, 309–316 (2012). 22176306 10.3109/14992027.2011.639812

[42] Prado-Gutierrez, P. *et al.* Habituation of auditory steady state responses evoked by amplitude-modulated acoustic signals in rats. *Audiol. Res.* **5**, 113 (2015). 26557360 10.4081/audiores.2015.113 PMC4627118

[43] Venkataraman, Y. & Bartlett, E. L. Postnatal development of auditory central evoked responses and thalamic cellular properties. *Dev. Neurobiol.* **74**, 541–555 (2014). 24214269 10.1002/dneu.22148

[44] Hörpel, S. G. & Firzlaff, U. Post-natal development of the envelope following response to amplitude modulated sounds in the bat phyllostomus discolor. *Hear. Res.* **388**, 107904 (2020). 32028065 10.1016/j.heares.2020.107904

[45] Artieda, J. *et al.* Potentials evoked by chirp-modulated tones: A new technique to evaluate oscillatory activity in the auditory pathway. *Clin. Neurophysiol.* **115**, 699–709 (2004). 15036066 10.1016/j.clinph.2003.10.021

[46] Purcell, D. W., John, S. M., Schneider, B. A. & Picton, T. W. Human temporal auditory acuity as assessed by envelope following responses. *J. Acoust. Soc. Am.* **116**, 3581–3593 (2004). 15658709 10.1121/1.1798354

[47] Riquelme, R., Kuwada, S., Filipovic, B., Hartung, K. & Leonard, G. Optimizing the stimuli to evoke the amplitude modulation following response (AMFR) in neonates. *Ear Hear.* **27**, 104–119 (2006). 16518139 10.1097/01.aud.0000201857.99240.24

[48] Konoike, N. *et al.* Comparison of non-invasive, scalp-recorded auditory steady-state responses in humans, rhesus monkeys, and common marmosets. *Sci. Rep.* **12**, 1–13 (2022). 35654875 10.1038/s41598-022-13228-8 PMC9163194

[49] Burton, M. J., Cohen, L. T., Rickards, F. W., McNally, K. I. & Clark, G. M. Steady-state evoked potentials to amplitude modulated tones in the monkey. *Acta Otolaryngol.* **112**, 745–751 (1992). 1456028 10.3109/00016489209137469

[50] Linnenschmidt, M., Wahlberg, M. & Damsgaard Hansen, J. The modulation rate transfer function of a harbour porpoise (*Phocoena phocoena*). *J. Compar. Physiol. A* **199**, 115–126 (2013). 10.1007/s00359-012-0772-8 23149551

[51] Pérez-Alcázar, M. *et al.* Chirp-evoked potentials in the awake and anesthetized rat. A procedure to assess changes in cortical oscillatory activity. *Exp. Neurol.* **210**, 144–153 (2008). 18177639 10.1016/j.expneurol.2007.10.017

[52] Prado-Gutiérrez, P. *et al.* A method for tracking the time evolution of steady-state evoked potentials. *J. Vis. Exp.* **1**, e59898 (2019). 10.3791/59898 PMC7055073 31180347

[53] Pauli-Magnus, D. *et al.* Detection and differentiation of sensorineural hearing loss in mice using auditory steady-state responses and transient auditory brainstem responses. *Neuroscience* **149**, 673–684 (2007). 17869440 10.1016/j.neuroscience.2007.08.010

[54] Li, Z. *et al.* Laminar profile of auditory steady-state response in the auditory cortex of awake mice. *Front. Syst. Neurosci.* **15**, 636395 (2021). 33815073 10.3389/fnsys.2021.636395 PMC8017131

[55] Mäkelä, J., Karmos, G., Molnar, M., Csepe, V. & Winkler, I. Steady-state responses from the cat auditory cortex. *Hear. Res.* **45**, 41–50 (1990). 2345117 10.1016/0378-5955(90)90181-N

[56] Ottaviani, F. *et al.* Auditory steady-state responses in the rabbit. *Audiology* **29**, 212–218 (1990). 2222290 10.3109/00206099009072852

[57] Dolphin, W. F., Chertoff, M. E. & Burkard, R. Comparison of the envelope following response in the Mongolian gerbil using two-tone and sinusoidally amplitude-modulated tones. *J. Acoust. Soc. Am.* **96**, 2225–2234 (1994). 7963035 10.1121/1.411382

[58] Szalda, K. & Burkard, R. The effects of nembutal anesthesia on the auditory steady-state response (ASSR) from the inferior colliculus and auditory cortex of the chinchilla. *Hear. Res.* **203**, 32–44 (2005). 15855028 10.1016/j.heares.2004.11.014

[59] Johnson, B. W., Weinberg, H., Ribary, U., Cheyne, D. O. & Ancill, R. Topographic distribution of the 40 Hz auditory evoked-related potential in normal and aged subjects. *Brain Topogr.* **1**, 117–121 (1988). 3275115 10.1007/BF01129176

[60] Roß, B., Borgmann, C., Draganova, R., Roberts, L. E. & Pantev, C. A high-precision magnetoencephalographic study of human auditory steady-state responses to amplitude-modulated tones. *J. Acoust. Soc. Am.* **108**, 679–691 (2000). 10955634 10.1121/1.429600

[61] Reyes, S. A. *et al.* Pet imaging of the 40 Hz auditory steady state response. *Hear. Res.* **194**, 73–80 (2004). 15276678 10.1016/j.heares.2004.04.001

[62] Kozono, N. *et al.* Auditory steady state response; nature and utility as a translational science tool. *Sci. Rep.* **9**, 1–10 (2019). 31186500 10.1038/s41598-019-44936-3 PMC6560088

[63] Musall, S., Von Pföstl, V., Rauch, A., Logothetis, N. K. & Whittingstall, K. Effects of neural synchrony on surface eeg. *Cereb. Cortex* **24**, 1045–1053 (2014). 23236202 10.1093/cercor/bhs389

[64] Vohs, J. L. *et al.* Gabaergic modulation of the 40 Hz auditory steady-state response in a rat model of schizophrenia. *Int. J. Neuropsychopharmacol.* **13**, 487–497 (2010). 19627651 10.1017/S1461145709990307 PMC2882653

[65] Sivarao, D. V. The 40-Hz auditory steady-state response: A selective biomarker for cortical nmda function. *Ann. N. Y. Acad. Sci.* **1344**, 27–36 (2015). 25809615 10.1111/nyas.12739

[66] McFadden, K. L. *et al.* Test-retest reliability of the 40 Hz eeg auditory steady-state response. *PLoS ONE* **9**, e85748 (2014). 24465679 10.1371/journal.pone.0085748 PMC3899078

[67] Marguet, S. L. & Harris, K. D. State-dependent representation of amplitude-modulated noise stimuli in rat auditory cortex. *J. Neurosci.* **31**, 6414–6420 (2011). 21525282 10.1523/JNEUROSCI.5773-10.2011 PMC3099304

[68] Herdman, A. T. *et al.* Intracerebral sources of human auditory steady-state responses. *Brain Topogr.* **15**, 69–86 (2002). 12537303 10.1023/A:1021470822922

[69] Mäkelä, J. & Hari, R. Evidence for cortical origin of the 40 Hz auditory evoked response in man. *Electroencephalogr. Clin. Neurophysiol.* **66**, 539–546 (1987). 2438120 10.1016/0013-4694(87)90101-5

[70] Hari, R., Hämäläinen, M. & Joutsiniemi, S.-L. Neuromagnetic steady-state responses to auditory stimuli. *J. Acoust. Soc. Am.* **86**, 1033–1039 (1989). 2794240 10.1121/1.398093

[71] Pantev, C., Roberts, L. E., Elbert, T., Rob, B. & Wienbruch, C. Tonotopic organization of the sources of human auditory steady-state responses. *Hear. Res.* **101**, 62–74 (1996). 8951433 10.1016/S0378-5955(96)00133-5

[72] Hutcheon, B. & Yarom, Y. Resonance, oscillation and the intrinsic frequency preferences of neurons. *Trends Neurosci.* **23**, 216–222 (2000). 10782127 10.1016/S0166-2236(00)01547-2

[73] Kuwada, S. & Batra, R. Coding of sound envelopes by inhibitory rebound in neurons of the superior olivary complex in the unanesthetized rabbit. *J. Neurosci.* **19**, 2273–2287 (1999). 10066278 10.1523/JNEUROSCI.19-06-02273.1999 PMC6782550

[74] Wang, X.-J. Neurophysiological and computational principles of cortical rhythms in cognition. *Physiol. Rev.* **90**, 1195–1268 (2010). 20664082 10.1152/physrev.00035.2008 PMC2923921

[75] Griskova-Bulanova, I., Sveistyte, K. & Bjekic, J. Neuromodulation of gamma-range auditory steady-state responses: A scoping review of brain stimulation studies. *Front. Syst. Neurosci.* **14**, 41 (2020). 32714158 10.3389/fnsys.2020.00041 PMC7344212

[76] Sun, Y. *et al.* Gamma oscillations in schizophrenia: Mechanisms and clinical significance. *Brain Res.* **1413**, 98–114 (2011). 21840506 10.1016/j.brainres.2011.06.065

[77] Williams, S. & Boksa, P. Gamma oscillations and schizophrenia. *J. Psychiatry Neurosci.* **35**, 75 (2010). 20184803 10.1503/jpn.100021 PMC2834788

[78] Shin, Y.-W., Odonnell, B. F., Youn, S. & Kwon, J. S. Gamma oscillation in schizophrenia. *Psychiatry Investig.* **8**, 288 (2011). 22216037 10.4306/pi.2011.8.4.288 PMC3246135

[79] Moran, L. V. & Hong, L. E. High vs low frequency neural oscillations in schizophrenia. *Schizophr. Bull.* **37**, 659–663 (2011). 21653278 10.1093/schbul/sbr056 PMC3122299

[80] Jefferys, J. G., Traub, R. D. & Whittington, M. A. Neuronal networks for induced 40 Hz rhythms. *Trends Neurosci.* **19**, 202–208 (1996). 8723208 10.1016/S0166-2236(96)10023-0

[81] Whittington, M. A., Traub, R. D., Kopell, N., Ermentrout, B. & Buhl, E. H. Inhibition-based rhythms: Experimental and mathematical observations on network dynamics. *Int. J. Psychophysiol.* **38**, 315–336 (2000). 11102670 10.1016/S0167-8760(00)00173-2

[82] Studer, F. & Barkat, T. R. Inhibition in the auditory cortex. *Neurosci. Biobehav. Rev.* **132**, 61–75 (2022). 34822879 10.1016/j.neubiorev.2021.11.021

[83] Frandolig, J. E. *et al.* The synaptic organization of layer 6 circuits reveals inhibition as a major output of a neocortical sublamina. *Cell Rep.* **28**, 3131–3143 (2019). 31533036 10.1016/j.celrep.2019.08.048 PMC6941480

[84] Mitani, A. & Shimokouchi, M. Neuronal connections in the primary auditory cortex: An electrophysiological study in the cat. *J. Compar. Neurol.* **235**, 417–429 (1985). 10.1002/cne.902350402 2987316

[85] Read, H. L., Winer, J. A. & Schreiner, C. E. Functional architecture of auditory cortex. *Curr. Opin. Neurobiol.* **12**, 433–440 (2002). 12139992 10.1016/S0959-4388(02)00342-2

[86] Prieto, J. J. & Winer, J. A. Layer vi in cat primary auditory cortex: Golgi study and sublaminar origins of projection neurons. *J. Compar. Neurol.* **404**, 332–358 (1999). 10.1002/(SICI)1096-9861(19990215)404:3<332::AID-CNE5>3.0.CO;2-R 9952352

[87] Ahmed, B., Anderson, J. C., Douglas, R. J., Martin, K. A. & Nelson, J. C. Polyneuronal innervation of spiny stellate neurons in cat visual cortex. *J. Compar. Neurol.* **341**, 39–49 (1994). 10.1002/cne.903410105 8006222

[88] Lee, C. C., Imaizumi, K., Schreiner, C. E. & Winer, J. A. Concurrent tonotopic processing streams in auditory cortex. *Cereb. Cortex* **14**, 441–451 (2004). 15028648 10.1093/cercor/bhh006

[89] Lee, C. C. & Sherman, S. M. Synaptic properties of thalamic and intracortical inputs to layer 4 of the first-and higher-order cortical areas in the auditory and somatosensory systems. *J. Neurophysiol.* **100**, 317–326 (2008). 18436628 10.1152/jn.90391.2008 PMC2493493

[90] Lee, C. C. & Sherman, S. M. Glutamatergic inhibition in sensory neocortex *Cereb. Cortex.* **19**(10), 2281–2289 (2009). 10.1093/cercor/bhn246 PMC2742591 19176638

[91] Sullivan, E. M., Timi, P., Hong, L. E. & Odonnell, P. Effects of nmda and gaba-a receptor antagonism on auditory steady-state synchronization in awake behaving rats. *Int. J. Neuropsychopharmacol.* **18**, 1 (2015). 10.1093/ijnp/pyu118 PMC4540097 25556198

[92] Borg, E. Auditory thresholds in rats of different age and strain. A behavioral and electrophysiological study. *Hear. Res.* **8**, 101–115 (1982). 7142038 10.1016/0378-5955(82)90069-7

[93] Rodríguez Valiente, A., Trinidad, A., García Berrocal, J., Górriz, C. & Ramírez Camacho, R. Extended high-frequency (9–20 kHz) audiometry reference thresholds in 645 healthy subjects. *Int. J. Audiol.* **53**, 531–545 (2014). 24749665 10.3109/14992027.2014.893375

