# Supplementary material for: Network resonance and the auditory steady state response
Source: Sci Rep. 2024 Jul 22;14:16799. doi: 10.1038/s41598-024-66697-4 (PMC11263589; doi:10.1038/s41598-024-66697-4)
Supplement: Supplementary file 1 — Supplementary Information. [file 41598_2024_66697_MOESM1_ESM.pdf]

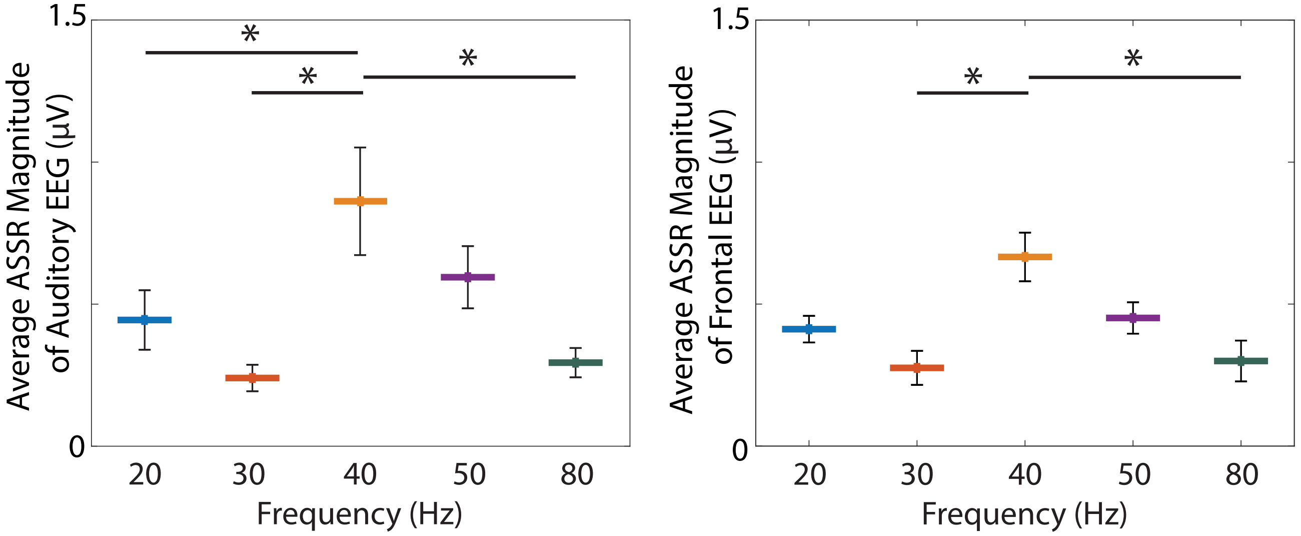

Figure S1. Average ASSR calculated from (left panel) auditory and (right panel) frontal EEG signals. Thick lines indicate mean values, and the height of black lines indicate 95% confidence intervals. Asterisks indicate significant differences in pairwise comparisons to the 40Hz AM condition ($p < 0.05$).

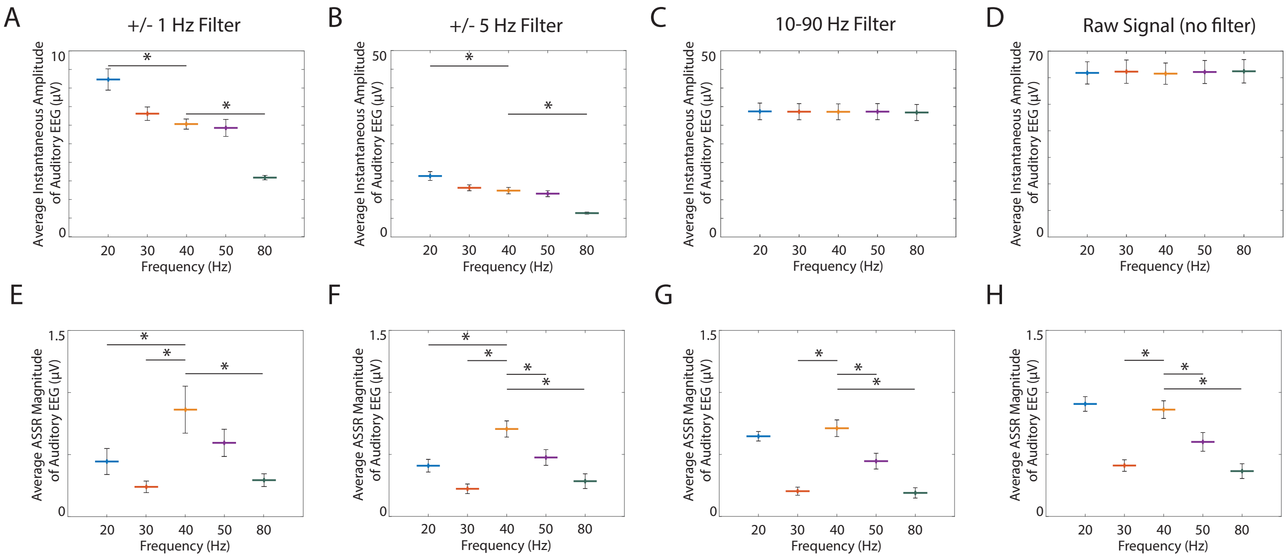

Figure S2. Average instantaneous amplitude (IA) across AM frequencies given A) +/- 1 Hz filter, B) +/- 5 Hz filter, C) a 10-90 Hz filter, or D) no filter of the auditory EEG before calculation of the IA. Modulation rate transfer functions depicting the average ASSR across AM frequencies given E) +/- 1 Hz filter, F) +/- 5 Hz filter, G) a 10-90 Hz filter, or H) no filter of the auditory EEG before calculation of the ASSR. In the case of no filter, the average signal of a previous cycle was subtracted from each cycle before calculation of the ASSR. We performed a repeated measures ANOVA across AM frequency conditions, with a Tukey's Honestly Significant Difference Test for individual comparisons to the 40 Hz AM condition. Asterisks indicate significant differences in pairwise comparisons to the 40 Hz AM condition ($p < 0.05$).

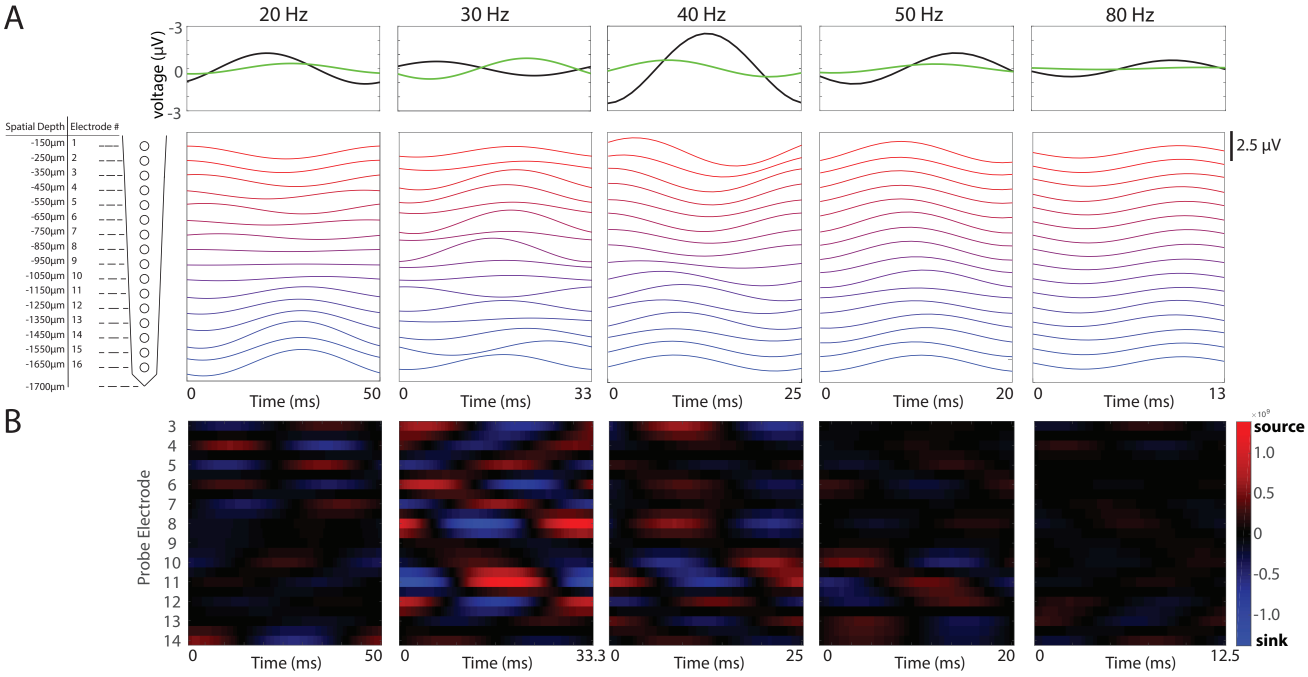

Figure S3. A)One-cycle averages across a single recording session, shown for each of the recording electrodes (green = frontal EEG; black = auditory EEG; red to blue shades = probe electrode channels from superficial to deep locations). One cycle averages were obtained by first filtering +/- 1 Hz surrounding the auditory stimulus frequency and then averaging signals within an auditory stimulus cycle period. Average signals are spread vertically to indicate their spatial location along the probe. Probe diagram indicating approximate depth is shown on the left, and a y-axis scale bar is shown on the right. B) Corresponding current source density for the one-cycle averages shown in A.

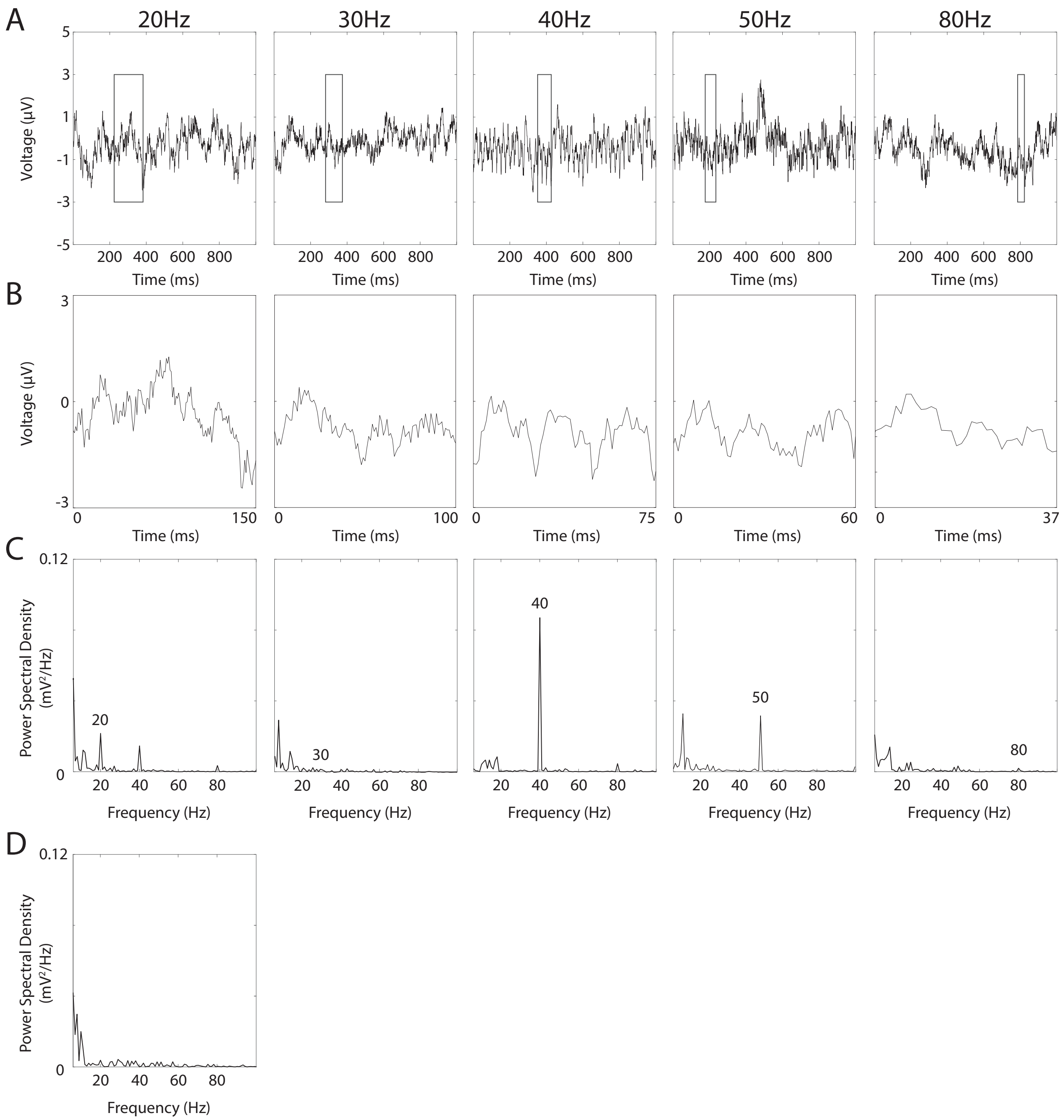

Figure S4. Characteristics of the rat ASSR. Each column represents a different AM frequency (from left to right: 20Hz, 30Hz, 40Hz, 50Hz, and 80Hz). A) Average of 1 s epochs of the raw auditory EEG signal, averaged first across 1 s segments of a given AM frequency block, and then averaged again across all recording sessions. B) Enlarged three cycle segment of A, corresponding to the time interval outlined in the black box. C) Power spectral density estimates of the 1s averages in A. D) Power spectral density estimates of the average raw auditory EEG signal during the baseline (with no auditory stimulus), averaged first across 1 s segments of the baseline and then averaged across all recording sessions. Statistical comparisons of the average power at the AM frequency during baseline and auditory stimulus conditions revealed significantly higher 40 and 80 Hz power during stimulus conditions (paired t-test, d.f. = 13, p = 0.03 for 40Hz, p = 0.05 for 80 Hz). We used the power at the AM frequency (e.g. 20Hz) minus the average power of four non-adjacent surrounding frequencies (e.g. 17, 18, 22, and 23 Hz) and then divided by the standard deviation of the four surrounding frequencies for statistical comparisons.

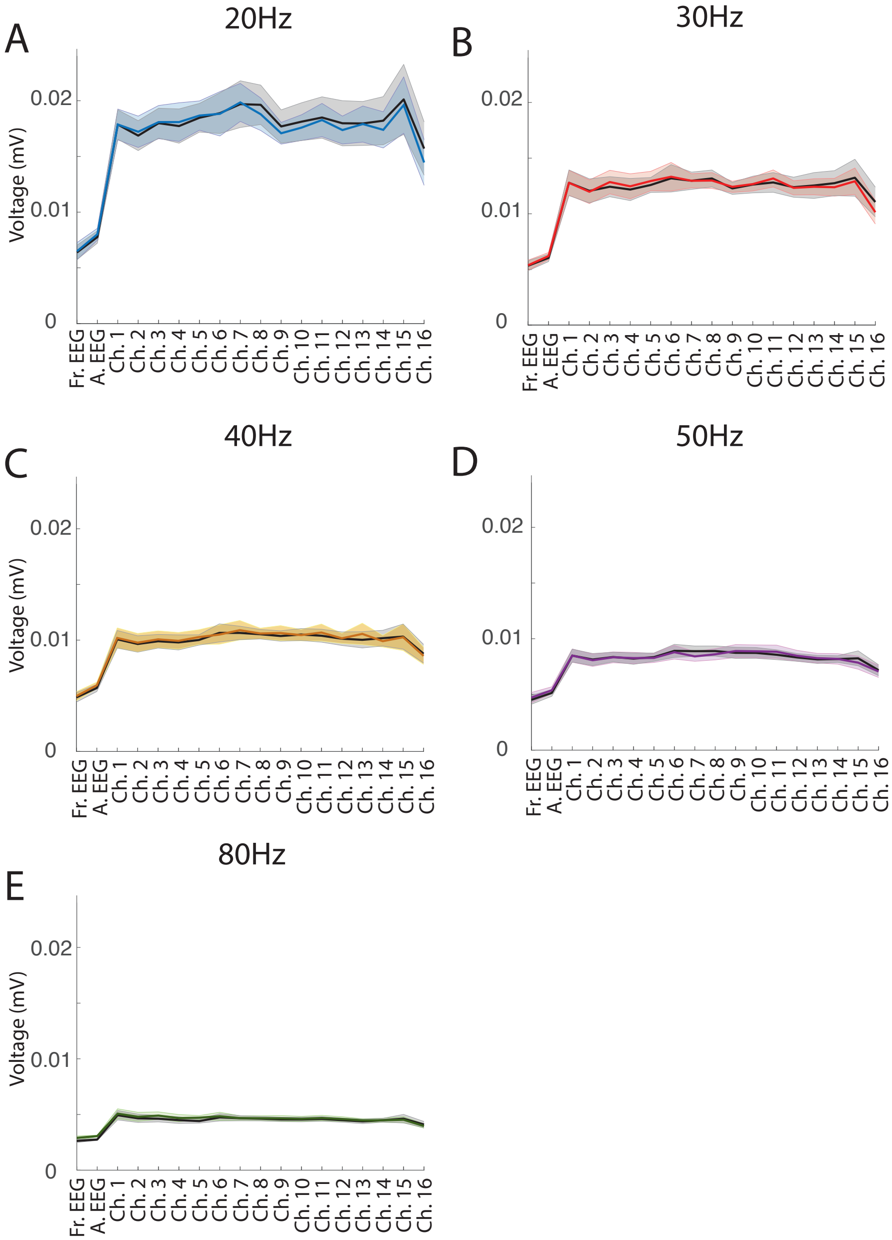

Figure S5. Average instantaneous amplitude (IA) of oscillations at each stimulus frequency during baseline (black lines) and sub-sampled auditory stimulus (colored lines) conditions. Each point on a line indicates the average IA of the filtered signal (+/- 1Hz surrounding the auditory stimulus frequency) across recording sessions, calculated separately for each recording electrode. Shaded areas surrounding each line indicate the standard error of the mean. Each plot corresponds to a different AM frequency condition: A) 20Hz (blue), B) 30Hz (red), C) 40Hz (yellow), D) 50Hz (purple), and E) 80Hz (green). We performed individual paired t-tests between baseline and stimulus conditions for each electrode (d.f. = 10 for Frontal EEG, d.f. = 13 for Auditory EEG, and d.f. = 16 for probe electrodes). Significant increases in the IA of 20Hz ($p = 0.03$), 50Hz ($p = 0.02$), and 80Hz ($p = 0.0003$) oscillations were found for the auditory EEG electrode, and significant increases in the IA of 80Hz oscillations were found for the frontal EEG electrode ($p = 0.003$).

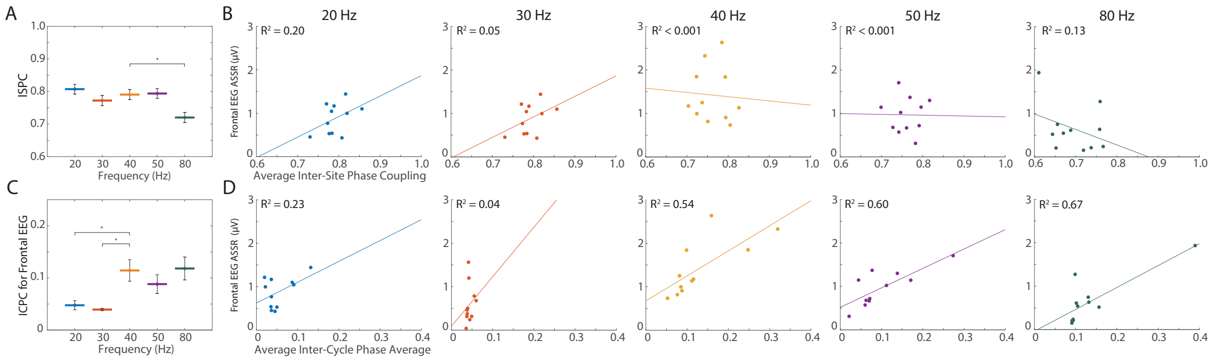

Figure S6. Relationship between ASSR calculated from the frontal EEG, inter-site phase coupling (ISPC), and inter-cycle phase coupling (ICPC). A) Average ISPC values during each AM frequency condition. Thick lines indicate mean values, and the height of black lines indicate 95% confidence intervals. Asterisks indicate significant differences in pairwise comparisons to the 40Hz AM condition ($p < 0.05$). B) ASSR from the frontal EEG as a function of the ISPC values, shown separately for each AM frequency condition. Each dot corresponds to a different recording session, and each panel indicates a different AM frequency condition. Corresponding R^2 values for the lines of best fit are shown in the upper left. C) Average ICPC values during each AM frequency condition, for signals acquired from the frontal EEG. Thick lines indicate mean values, and the height of black lines indicate 95% confidence intervals. Asterisks indicate significant differences in pairwise comparisons to the 40Hz AM condition ($p < 0.05$). D) ASSR from the frontal EEG as a function of the ICPC values, shown separately for each AM frequency condition. Each dot corresponds to a different recording session, and each panel indicates a different AM frequency condition. Corresponding R^2 values for the lines of best fit are shown in the upper left.

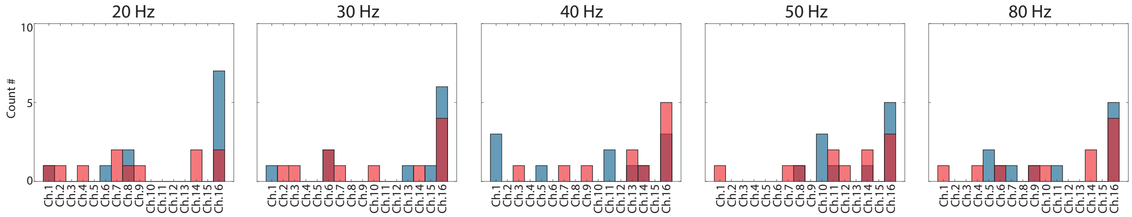

Figure S7. Modeling results assessing filtered probe signal (filtered +/- 1Hz surrounding the auditory stimulus frequency) covariance with the filtered frontal EEG signal. A) Histogram indicating the spatial location of the channels that were the most predictive (blue) and the second most predictive (red) of the filtered frontal EEG.

Comparison of ASSR Across Stimulus Conditions					
	RM Anova	20Hz	30Hz	50Hz	80Hz
Frontal EEG	$p = 0.0008$, $F(4,10) = 5.9$	N.S.	$p = 9.41E-4$	N.S.	$p = 0.002$
Auditory EEG	$p = 8.57E-6$, $F(4,13) = 9.4$	$p = 0.006$	$p = 1.81E-5$	N.S.	$p = 9.19E-5$
Electrode 1	$p < 1.00E-9$, $F(4,16) = 9.79$	N.S.	$p = 2.39E-4$	$p = 0.013$	$p = 2.20E-6$
Electrode 2	$p < 1.00E-9$, $F(4,16) = 8.15$	N.S.	$p = 0.001$	$p = 0.013$	$p = 1.91E-5$
Electrode 3	$p = 0.0001$, $F(4,16) = 6.92$	N.S.	$p = 0.009$	N.S.	$p = 2.00E-4$
Electrode 4	$p = 0.0001$, $F(4,16) = 7.1$	N.S.	$p = 0.002$	N.S.	$p = 1.84E-4$
Electrode 5	$p < 1.00E-9$, $F(4,16) = 7.1$	N.S.	$p = 0.001$	N.S.	$p = 1.51E-4$
Electrode 6	$p < 1.00E-9$, $F(4,16) = 8.86$	N.S.	$p = 0.006$	$p = 0.033$	$p = 7.30E-5$
Electrode 7	$p < 1.00E-9$, $F(4,16) = 9.88$	N.S.	$p = 0.001$	$p = 0.029$	$p = 8.36E-5$
Electrode 8	$p < 1.00E-9$, $F(4,16) = 10.11$	N.S.	$p = 0.005$	N.S.	$p = 3.46E-5$
Electrode 9	$p < 1.00E-9$, $F(4,16) = 8.75$	N.S.	$p = 0.001$	$p = 0.044$	$p = 1.24E-4$
Electrode 10	$p < 1.00E-9$, $F(4,16) = 12.67$	N.S.	$p = 3.46E-4$	$p = 0.040$	$p = 9.54E-6$
Electrode 11	$p < 1.00E-9$, $F(4,16) = 11.02$	N.S.	$p = 3.25E-4$	$p = 0.010$	$p = 1.47E-5$
Electrode 12	$p < 1.00E-9$, $F(4,16) = 12.1$	N.S.	$p = 1.94E-4$	$p = 0.013$	$p = 4.15E-6$
Electrode 13	$p < 1.00E-9$, $F(4,16) = 12.28$	N.S.	$p = 7.57E-5$	$p = 0.004$	$p = 1.56E-6$
Electrode 14	$p < 1.00E-9$, $F(4,16) = 13.89$	N.S.	$p = 9.85E-5$	$p = 0.009$	$p = 5.95E-7$
Electrode 15	$p < 1.00E-9$, $F(4,16) = 16.49$	N.S.	$p = 1.11E-5$	$p = 7.2E-4$	$p = 6.43E-8$
Electrode 16	$p < 1.00E-9$, $F(4,16) = 12.47$	N.S.	$p = 7.11E-5$	N.S.	$p = 1.89E-5$

Table S1. We performed a repeated measures ANOVA across AM frequency conditions for each electrode, with a Tukey's Honestly Significant Difference Test for individual pairwise comparisons to the 40Hz AM condition.

Comparisons of ASSR Magnitude Across Baseline and Stimulus Conditions					
	20Hz	30Hz	40Hz	50Hz	80Hz
Frontal EEG	N.S.	N.S.	$p = 0.004$, $t = 13.86$	$p = 0.0082$, $t = 10.81$	N.S.
Auditory EEG	N.S.	N.S.	$p = 0.0017$, $t = 15.43$	$p = 0.0065$, $t = 10.48$	$p = 3.86E-4$, $t = 22.4747$
Electrode 1	N.S.	N.S.	$p = 0.0048$, $t = 10.6877$	N.S.	$p = 1.05E-6$, $t = 57.9579$
Electrode 2	N.S.	N.S.	$p = 0.0079$, $t = 9.2174$	$p = 0.0097$, $t = 8.6151$	$p = 3.17E-8$, $t = 98.1152$
Electrode 3	N.S.	N.S.	N.S.	$p = 0.0090$, $t = 8.8347$	$p = 6.67E-7$, $t = 62.2085$
Electrode 4	N.S.	N.S.	$p = 0.0037$, $t = 11.4078$	$p = 0.0076$, $t = 9.3179$	$p = 2.76E-6$, $t = 49.6585$
Electrode 5	N.S.	N.S.	$p = 5.76E-4$, $t = 18.3025$	N.S.	$p = 7.38E-6$, $t = 42.1911$
Electrode 6	N.S.	N.S.	$p = 3.63E-5$, $t = 31.9046$	N.S.	$p = 2.49E-7$, $t = 72.3092$
Electrode 7	N.S.	N.S.	$p = 4.94E-4$, $t = 18.9438$	N.S.	$p = 5.22E-7$, $t = 64.5960$
Electrode 8	N.S.	N.S.	$p = 7.22E-4$, $t = 17.3887$	N.S.	$p = 6.43E-7$, $t = 62.5541$
Electrode 9	N.S.	N.S.	$p = 4.33E-4$, $t = 19.4996$	N.S.	$p = 1.51E-6$, $t = 54.7319$
Electrode 10	N.S.	N.S.	$p = 3.36E-4$, $t = 20.5893$	N.S.	$p = 2.04E-6$, $t = 52.1413$
Electrode 11	N.S.	N.S.	$p = 6.05E-4$, $t = 18.1063$	N.S.	$p = 1.34E-6$, $t = 55.7635$
Electrode 12	N.S.	N.S.	$p = 7.92E-4$, $t = 17.0271$	N.S.	$p = 3.64E-6$, $t = 47.4699$
Electrode 13	N.S.	N.S.	$p = 7.66E-4$, $t = 17.1573$	N.S.	$p = 3.21E-6$, $t = 48.4583$
Electrode 14	N.S.	N.S.	$p = 0.0012$, $t = 15.3031$	N.S.	$p = 1.40E-5$, $t = 37.8224$
Electrode 15	N.S.	N.S.	$p = 0.0067$, $t = 9.7088$	N.S.	$p = 2.30E-6$, $t = 51.1548$
Electrode 16	N.S.	N.S.	$p = 0.0024$, $t = 12.9264$	$p = 0.0063$, $t = 9.8941$	$p = 4.28E-5$, $t = 30.9497$

Table S2. We performed individual paired t-tests between baseline and stimulus conditions for each electrode (d.f. = 10 for Frontal EEG, d.f. = 13 for Auditory EEG, and d.f. = 16 for probe electrodes).

Comparisons of ICPC Values Across Stimulus Conditions					
	RM Anova	20Hz PW	30Hz PW	50Hz PW	80Hz PW
Frontal EEG	$p = 4.38E-5, F(4,10) = 8.55$	$p = 0.005$	$p = 0.001$	N.S.	N.S.
Auditory EEG	$p = 1.53E-5, F(4,13) = 8.88$	$p = 9.39E-4$	$p = 3.06E-4$	N.S.	N.S.
Electrode 1	$p = 2.19E-7, F(4,16) = 12.1$	$p = 4.72E-4$	$p = 0.005$	N.S.	N.S.
Electrode 2	$p = 2.46E-6, F(4,16) = 9.97$	$p = 0.002$	$p = 0.005$	N.S.	N.S.
Electrode 3	$p < 1.00E-9, F(4,16) = 10.1$	$p = 0.004$	$p = 0.02$	N.S.	N.S.
Electrode 4	$p = 1.50E-6, F(4,16) = 10.39$	$p = 0.01$	$p = 0.015$	N.S.	N.S.
Electrode 5	$p = 2.39E-7, F(4,16) = 12.03$	$p = 0.015$	$p = 0.007$	N.S.	N.S.
Electrode 6	$p < 1.00E-9, F(4,16) = 9.37$	$p = 0.014$	$p = 0.01$	N.S.	N.S.
Electrode 7	$p < 1.00E-9, F(4,16) = 10.64$	$p = 0.021$	$p = 0.027$	N.S.	N.S.
Electrode 8	$p < 1.00E-9, F(4,16) = 9.6$	$p = 0.044$	$p = 0.038$	N.S.	N.S.
Electrode 9	$p < 1.00E-9, F(4,16) = 12.62$	$p = 0.041$	$p = 0.01$	N.S.	N.S.
Electrode 10	$p < 1.00E-9, F(4,16) = 12.61$	$p = 0.024$	$p = 0.017$	N.S.	$p = 0.045$
Electrode 11	$p < 1.00E-9, F(4,16) = 14.26$	$p = 0.005$	$p = 0.004$	N.S.	N.S.
Electrode 12	$p < 1.00E-9, F(4,16) = 17.87$	$p = 0.007$	$p = 0.003$	N.S.	$p = 0.009$
Electrode 13	$p < 1.00E-9, F(4,16) = 16.41$	$p = 0.002$	$p = 8.68E-4$	N.S.	N.S.
Electrode 14	$p < 1.00E-9, F(4,16) = 16.93$	$p = 0.004$	$p = 8.41E-4$	N.S.	$p = 0.043$
Electrode 15	$p < 1.00E-9, F(4,16) = 21.54$	$p = 3.74E-5$	$p = 1.98E-5$	$p = 0.006$	N.S.
Electrode 16	$p < 1.00E-9, F(4,16) = 14.76$	$p = 0.002$	$p = 5.77E-5$	N.S.	N.S.

Table S3. We performed a repeated measures ANOVA across AM frequency conditions for each electrode, with a Tukey's Honestly Significant Difference Test for individual pairwise comparisons to the 40Hz AM condition.

ICPC Model Results					
	20Hz	30Hz	40Hz	50Hz	80Hz
F. EEG	N.S.	N.S.	$p = 0.01, t = 3.28$	$p = 0.005, t = 3.68$	$p = 0.002, t = 4.25$
A. EEG	$p = 5.91E-10, t = 17.66$	$p = 1.16E-4, t = 5.60$	$p = 4.43E-9, t = 14.83$	$p = 3.92E-6, t = 7.97$	$p = 1.14E-5, t = 7.17$
Electrode 1	N.S.	$p = 0.026, t = 2.47$	$p = 4.73E-6, t = 6.94$	$p = 1.21E-8, t = 11.13$	$p = 3.95E-5, t = 5.73$
Electrode 2	N.S.	N.S.	$p = 6.58E-7, t = 8.18$	$p = 1.23E-8, t = 11.11$	$p = 4.46E-5, t = 5.67$
Electrode 3	N.S.	$p = 0.005, t = 3.28$	$p = 2.84E-7, t = 8.74$	$p = 4.32E-9, t = 12.00$	$p = 4.66E-6, t = 6.95$
Electrode 4	N.S.	$p = 0.021, t = 2.58$	$p = 9.72E-7, t = 7.92$	$p = 2.81E-8, t = 10.44$	$p = 3.17E-5, t = 5.85$
Electrode 5	N.S.	$p = 0.032, t = 2.37$	$p = 1.95E-6, t = 7.48$	$p = 3.22E-9, t = 12.26$	$p = 3.62E-5, t = 5.78$
Electrode 6	N.S.	$p = 0.004, t = 3.37$	$p = 1.18E-5, t = 6.40$	$p = 8.04E-8, t = 9.64$	$p = 3.67E-5, t = 5.77$
Electrode 7	N.S.	$p = 0.01, t = 2.91$	$p = 9.66E-9, = 11.31$	$p = 1.09E-9, t = 13.26$	$p = 3.67E-5, t = 5.77$
Electrode 8	N.S.	$p = 0.001, t = 3.97$	$p = 1.23E-7, t = 9.33$	$p = 8.09E-9, t = 11.46$	$p = 1.51E-4, t = 5.02$
Electrode 9	N.S.	$p = 0.006, t = 3.18$	$p = 4.73E-8, t = 10.04$	$p = 3.08E-11, t = 17.07$	$p = 1.37E-4, t = 5.08$
Electrode 10	N.S.	$p = 0.031, t = 2.38$	$p = 5.73E-8, t = 9.90$	$p < 1.00E-9, t = 13.73$	$p = 5.04E-6, t = 6.90$
Electrode 11	N.S.	N.S.	$p = 5.15E-9, t = 11.85$	$p < 1.00E-9, tstat = 15.40$	$p = 1.22E-4, t = 5.14$
Electrode 12	N.S.	$p = 0.009, t = 3.00$	$p < 1.00E-9, t = 15.16$	$p < 1.00E-9, t = 16.53$	$p = 3.60E-7, t = 8.58$
Electrode 13	N.S.	$p = 0.019, t = 2.64$	$p < 1.00E-9, t = 15.96$	$p < 1.00E-9, t = 18.02$	$p = 1.95E-8, t = 10.73$
Electrode 14	N.S.	N.S.	$p = 3.92E-8, t = 10.19$	$p < 1.00E-9, t = 14.38$	$p = 3.35E-8, t = 10.31$
Electrode 15	N.S.	N.S.	$p = 4.98E-7, t = 8.36$	$p < 1.00E-9, t = 11.51$	$p = 1.39E-7, t = 9.24$
Electrode 16	N.S.	N.S.	$p = 1.09E-5, t = 6.45$	$p = 6.07E-6, t = 6.79$	$p = 5.46E-6, t = 6.86$

Table S4. We report p-values for individual models relating the ASSR to average ICPC values for a given electrode and AM frequency condition.
